# Microbiological Spectrum and Antimicrobial Resistance Patterns in Hand Surgery Infections: A Monocentric Retrospective Study

**DOI:** 10.3390/pathogens15020183

**Published:** 2026-02-06

**Authors:** Lorenzo Drago, Fabiana Giarritiello, Deflorio Loredana, Luigi Regenburgh De La Motte, Francesca Carreras, Carmen Sommese, Giorgio Eugenio Pajardi, Luigi Troisi

**Affiliations:** 1 UOC Laboratory of Clinical Medicine with Specialized Areas, IRCCS MultiMedica, 20138 Milan, Italy; f.giarritiello@studenti.unimol.it (F.G.); loredana.deflorio@multimedica.it (D.L.); luigi.delamotte@multimedica.it (L.R.D.L.M.); francesca.carreras@multimedica.it (F.C.); carmen.sommese@multimedica.it (C.S.); 2 Department of Biomedical Health Sciences, University of Milan, 20133 Milan, Italy; 3 Department of Medicine and Health Sciences “V. Tiberio”, University of Molise, 86100 Campobasso, Italy; 4 Hand Surgery Unit, IRCCS MultiMedica, Sesto San Giovanni, 20099 Milan, Italy; giorgio.pajardi@unimi.it (G.E.P.)

**Keywords:** hand surgery infections, surgical site infections, antimicrobial resistance, trauma-related infections, implant-associated infections, cardiometabolic comorbidities

## Abstract

**Background:** Infections in hand surgery represent a clinically relevant complication, particularly in trauma-related procedures and in the presence of internal fixation devices. Data specifically addressing microbiological profiles and antimicrobial resistance patterns in hand surgery remain limited. **Methods:** A monocentric retrospective observational study was conducted, including 72 patients treated for hand surgery infections between January 2024 and June 2025. Microbiological isolates and antimicrobial susceptibility profiles were analyzed and stratified according to the clinical scenario, including trauma-related infections and infections associated with internal fixation devices. Monomicrobial and polymicrobial infections were evaluated separately. **Results:** Trauma-related infections accounted for 77.8% of cases, of which 64.3% were monomicrobial and 35.7% polymicrobial. Monomicrobial trauma-related infections were predominantly caused by *Staphylococcus aureus*, with methicillin resistance detected in 25.0% of cases. Polymicrobial trauma-related infections showed greater microbiological complexity but limited antimicrobial resistance. Infections associated with internal fixation devices represented 22.2% of cases and demonstrated a higher proportion of polymicrobial infections. Across all subgroups, no extended-spectrum beta-lactamase–producing Enterobacterales or carbapenem-resistant organisms were identified. **Conclusions:** This study fills an important evidence gap by characterizing pathogens and antimicrobial resistance in a dedicated hand surgery cohort, an area where published microbiological data remain limited compared with other orthopedic subspecialties. Hand surgery infections exhibit distinct microbiological and resistance profiles depending on the clinical scenario and microbial complexity. Despite frequent polymicrobial involvement, high-level antimicrobial resistance remains uncommon, supporting the value of local microbiological surveillance to guide empirical therapy.

## 1. Introduction

Hand surgery encompasses a wide spectrum of procedures, ranging from elective soft tissue interventions to complex trauma-related surgeries involving bones, tendons, joints, and implanted devices [1,2]. Owing to the unique anatomical characteristics of the hand, such as limited soft tissue coverage, multiple closed anatomical compartments, and the close proximity of structures essential for function, even localized infections may rapidly progress and lead to significant functional impairment [3]. Consequently, infections represent a particularly relevant complication in hand surgery, with potential long-term clinical, functional, and socioeconomic consequences [4].

Surgical site infections (SSIs) remain among the most frequent healthcare-associated infections worldwide [5]. According to the European Centre for Disease Prevention and Control (ECDC), SSIs account for approximately 20% of all healthcare-associated infections in hospitalized patients, with an overall incidence of around 1.6 per 100 surgical procedures across Europe (https://www.ecdc.europa.eu/en/healthcare-associated-infections, accessed on 21 January 2026). Although orthopaedic procedures are generally associated with lower SSI rates compared with abdominal or colorectal surgery, infections in orthopaedic and trauma surgery are particularly challenging due to the frequent involvement of implanted materials and the associated risk of persistent or chronic infection [6,7].

In the specific context of hand surgery, available epidemiological data are limited, and reported infection rates vary widely depending on the type of procedure and the degree of contamination [8]. Elective clean procedures of the hand, such as carpal tunnel release, are associated with very low infection rates, often below 1% [9]. In contrast, traumatic hand injuries—particularly open fractures and crush injuries—carry a substantially higher risk of infection. Studies focusing on open hand fractures have reported infection rates ranging from 5% to over 10%, especially in cases involving severe soft tissue damage and environmental contamination [10,11].

The microbiological spectrum of hand infections largely reflects skin flora, with *Staphylococcus aureus* being the most frequently isolated pathogen [12]. Coagulase-negative staphylococci, particularly *Staphylococcus epidermidis*, play a prominent role in infections associated with internal fixation devices and prosthetic implants due to their ability to form biofilms [13,14]. Streptococci and Gram-negative bacteria, including *Pseudomonas aeruginosa* and Enterobacterales, are more commonly isolated in contaminated wounds and trauma-related infections [15]. However, the relative distribution of pathogens may vary substantially according to the underlying clinical scenario and local epidemiology [16].

Antimicrobial resistance represents an additional challenge in the management of hand surgery infections. Methicillin-resistant *Staphylococcus aureus* (MRSA) has emerged as a relevant pathogen in musculoskeletal infections, with important implications for empirical antibiotic therapy [17]. In parallel, resistance among Gram-negative organisms—including multidrug resistance, extended-spectrum beta-lactamase (ESBL) production, and carbapenem resistance—has become a growing concern in hospital settings [18]. Italy is among the European countries with the highest burden of infections caused by multidrug-resistant organisms, further emphasizing the need for local surveillance data to support antimicrobial stewardship strategies (https://www.ecdc.europa.eu/en/publications-data/surveillance-antimicrobial-resistance-europe-2024-data, accessed on 21 January 2026).

Despite the clinical relevance of infections in hand surgery, most available microbiological and resistance data derive from broader orthopaedic or general surgical cohorts [19,20]. Dedicated studies focusing specifically on hand surgery infections are scarce, and few have systematically analyzed pathogen distribution and antimicrobial resistance patterns across different clinical scenarios, such as trauma-related infections, surgical site infections, and infections associated with internal fixation or prosthetic devices [4].

For these reasons, a detailed characterization of the microbiological spectrum and antimicrobial resistance patterns in hand surgery infections is warranted. The present monocentric retrospective study aims to map the distribution of causative microorganisms and antibiotic resistance profiles in a cohort of patients with hand surgery infections, stratified according to the underlying clinical scenario.

Hand surgery infections often occur in patients with systemic comorbidities, including cardiovascular disease, diabetes mellitus, and other metabolic disorders, which are known to impair immune response, wound healing, and infection outcomes.

The primary objective of the study is not to estimate infection incidence, but to provide a comprehensive microbiological and resistance profile of infected patients treated in a specialized hand surgery unit.

## 2. Materials and Methods

### 2.1. Study Design, Setting and Study Population

This monocentric retrospective observational study was conducted at the University Department of Hand Surgery & Rehabilitation, IRCCS MultiMedica Group, Milan, Italy, a tertiary referral center for hand and upper limb surgery. All patients treated for hand surgery infections during the study period (2024–2025) were retrospectively reviewed. Inclusion required the availability of at least one microbiological culture obtained during the infectious episode.

A total of 72 patients met the inclusion criteria and were included in the final analysis. Patients without available microbiological data were excluded. The resulting study population therefore represents a cohort of microbiologically confirmed infected patients.

Hand surgery–related infection was defined as a clinically suspected infection involving the hand, documented in the medical record and prompting therapeutic management (antimicrobial therapy and/or surgical intervention), with at least one microbiologically positive culture obtained from a clinically relevant specimen (e.g., purulent material, tissue, or intraoperative samples). Cultures obtained for surveillance purposes or without clinical evidence of infection were not included. When available, diagnosis was supported by local inflammatory signs and/or purulent drainage. Given the retrospective design, depth of infection (superficial vs. deep) was not systematically classified and was analyzed according to the information available in clinical documentation.

### 2.2. Clinical Classification and Data Collection

Infections were classified according to the underlying etiological scenario into trauma-related infections and infections associated with internal fixation devices or prosthetic implants. Trauma-related infections included open fractures, crush injuries, lacerations, bite wounds, and other contaminated traumatic events involving the hand. Due to the limited number of cases in specific trauma subtypes, no separate subgroup analysis was performed. Surgical site infection (SSI) status was recorded independently as an outcome-based classification according to CDC/NHSN criteria and may overlap with trauma-related or fixation-associated cases. Surgical site infections were defined in accordance with the Centers for Disease Control and Prevention/National Healthcare Safety Network (CDC/NHSN) criteria. Clinical, surgical, and microbiological data were extracted from electronic medical records and microbiology laboratory reports. Collected variables included demographic characteristics, clinical context of infection, presence of internal fixation devices or prosthetic material, microbiological isolates, antimicrobial susceptibility profiles, and the occurrence of polymicrobial infection.

### 2.3. Microbiological Analysis and Statistical Analysis

Microbiological data were obtained from routine culture reports generated during standard clinical practice. Isolated pathogens were classified as Gram-positive or Gram-negative bacteria according to standard laboratory criteria. Antimicrobial susceptibility testing and resistance categorization were performed in accordance with the European Committee on Antimicrobial Susceptibility Testing (EUCAST) guidelines. The analysis systematically included the assessment of methicillin resistance in *Staphylococcus aureus* and coagulase-negative staphylococci, multidrug resistance, extended-spectrum beta-lactamase (ESBL) production, and resistance to carbapenems. Polymicrobial infection was defined as the isolation of two or more distinct microorganisms from the same infectious episode. In polymicrobial infections, antimicrobial resistance analysis was based on the principal pathogen, defined as the microorganism showing the highest relative colony burden in culture and therefore considered microbiologically dominant compared with co-isolated organisms. For surgical site infections, the proportion of SSI cases within the infected cohort was recorded. Comorbidities were extracted from available medical documentation and were not collected through a standardized prospective registry. Hypertension was recorded as a previously documented diagnosis in the medical record.

## 3. Results

### 3.1. Study Population and Clinical Characteristics

A total of 72 patients with hand surgery–related infections were included in this monocentric retrospective study. Patient age ranged from 21 to 100 years, with a marked male predominance (73.6%, *n* = 53).

The demographic and clinical characteristics of the study population are summarized in Table 1. Trauma-related infections accounted for the majority of cases (77.8%, *n* = 56), while infections associated with internal fixation devices were observed in 22.2% of patients (*n* = 16). Surgical site infections (SSIs) were identified in 18.1% of cases (*n* = 13). SSI cases overlapped with both trauma-related and fixation-associated infections and are reported separately as an outcome classification.

Comorbidities were documented in a subset of patients. The most frequently reported conditions included cardiovascular disease (5.6%), diabetes mellitus (5.6%), smoking history (5.6%), and hypertension (4.2%). Less common comorbidities included chronic obstructive pulmonary disease, systemic autoimmune disorders, and malignancy. Several patients presented with more than one underlying condition.

Because the study was designed as a microbiological case series of infected patients and not as an incidence study, the total denominator of surgical procedures was not systematically collected, and reliable incidence estimates cannot be provided. The reported SSI proportion therefore reflects the distribution within the infected cohort rather than a population-level surgical incidence. The complete list of less frequent organisms is reported in Supplementary Table S1.

### 3.2. Microbiological Findings

#### 3.2.1. Trauma-Related Infections: Monomicrobial Versus Polymicrobial

Among the 72 patients included in the study, trauma-related infections accounted for 56 cases (77.8%). Within this subgroup, 36 infections (64.3%) were monomicrobial, while 20 cases (35.7%) were polymicrobial (Table 2).

Overall, trauma-related infections were predominantly sustained by Gram-positive bacteria, with *Staphylococcus aureus* representing the most frequently isolated pathogen across both monomicrobial and polymicrobial infections.

In monomicrobial trauma-related infections, *Staphylococcus aureus* was the leading pathogen, accounting for the majority of isolates, including both methicillin-susceptible and methicillin-resistant strains. Streptococcal species represented the second most frequent group, followed by Gram-negative bacilli such as *Pseudomonas aeruginosa* and *Escherichia coli*. A limited number of infections were caused by other Gram-positive organisms or less common pathogens. Overall, Gram-positive bacteria clearly predominated in monomicrobial trauma-related infections.

Polymicrobial trauma-related infections exhibited a more heterogeneous microbiological spectrum. These infections included combinations of Gram-positive cocci, Gram-negative bacilli, and anaerobic bacteria. Methicillin-susceptible *Staphylococcus aureus* remained the most frequently isolated organism, while Gram-negative pathogens such as *Pseudomonas aeruginosa* and Enterobacterales—including *Klebsiella oxytoca* and other species—were commonly identified. Enterococci and streptococci were also frequently involved. Anaerobic bacteria, particularly species of the genus *Prevotella*, were detected in a subset of cases.

Compared with monomicrobial infections, polymicrobial trauma-related infections demonstrated a greater contribution of Gram-negative and anaerobic organisms, although Gram-positive bacteria remained a consistent and prominent component of the microbiological profile.

#### 3.2.2. Infections Associated with Internal Fixation Devices: Monomicrobial Versus Polymicrobial

Among the 72 patients included in the study, 16 patients (22.2%) presented with infections associated with internal fixation devices (Table 3). Within this subgroup, 9 infections were monomicrobial (56.3% of fixation-related infections; 12.5% of the overall cohort), whereas 7 infections were polymicrobial (43.7% of fixation-related infections; 9.7% of the overall cohort).

Overall, infections associated with internal fixation devices were predominantly sustained by Gram-positive bacteria, with *Staphylococcus aureus* representing the most frequently isolated pathogen across both monomicrobial and polymicrobial infections.

In monomicrobial fixation-related infections, *Staphylococcus aureus* was the leading pathogen, accounting for the majority of isolates, including both methicillin-susceptible and methicillin-resistant strains. Streptococcal species were also identified, while Gram-negative bacteria were rarely involved, with *Pseudomonas aeruginosa* isolated in a single case. Overall, Gram-positive cocci clearly predominated in monomicrobial infections associated with internal fixation devices.

Polymicrobial fixation-related infections demonstrated a more heterogeneous microbiological profile. Although methicillin-susceptible *Staphylococcus aureus* remained the most frequently identified organism, additional pathogens included enterococci and other microorganisms grouped as “Others”, reflecting the presence of Gram-negative bacilli and anaerobic bacteria. Compared with monomicrobial cases, polymicrobial fixation-related infections showed a greater relative contribution of non–Gram-positive organisms, consistent with increased microbiological complexity in this subgroup.

### 3.3. Antimicrobial Resistance

Antimicrobial susceptibility profiles were analyzed for predominant bacterial pathogens isolated from trauma-related infections and infections associated with internal fixation devices. For trauma-related infections, resistance analysis included all isolates obtained from both monomicrobial and polymicrobial episodes, with polymicrobial infections evaluated based on the principal pathogen. The analysis specifically assessed methicillin resistance in *Staphylococcus aureus* and coagulase-negative staphylococci, multidrug resistance (MDR), fluoroquinolone resistance, extended-spectrum beta-lactamase (ESBL) production among Enterobacterales, and carbapenem resistance among Gram-negative bacteria. No ESBL-producing Enterobacterales or carbapenem-resistant organisms were identified in the study population.

#### 3.3.1. Antimicrobial Resistance of Predominant Bacterial Isolates in Trauma-Related Infections

Among trauma-related infections, *Staphylococcus aureus* was the most frequently isolated pathogen (*n* = 28). The antimicrobial resistance patterns of the predominant bacterial isolates are summarized in Table 4. Methicillin-resistant *S. aureus* (MRSA) accounted for 25.0% of isolates (7/28). All staphylococcal isolates remained fully susceptible to glycopeptides, with no resistance to vancomycin or teicoplanin detected. Resistance to ciprofloxacin and trimethoprim–sulfamethoxazole among *S. aureus* isolates was observed in 21.4% and 14.3% of cases, respectively. Multidrug resistance was uncommon and identified in one isolate (3.6%).

*Pseudomonas aeruginosa* represented the most frequent Gram-negative pathogen among trauma-related infections (*n* = 4). Resistance to piperacillin–tazobactam, third- and fourth-generation cephalosporins, and ciprofloxacin was detected in 25.0% of isolates. All *P. aeruginosa* isolates remained susceptible to carbapenems, with no carbapenem-resistant strains identified.

Overall, antimicrobial resistance among predominant bacterial isolates in trauma-related infections was limited, with preserved susceptibility to glycopeptides among Gram-positive cocci and to carbapenems among Gram-negative bacteria.

#### 3.3.2. Antimicrobial Resistance of Predominant Bacterial Isolates in Infections Associated with Internal Fixation Devices

In infections associated with internal fixation devices, *Staphylococcus aureus* was the predominant bacterial pathogen, accounting for the majority of isolates. Methicillin resistance was identified in a single *S. aureus* isolate.

All staphylococcal isolates remained fully susceptible to glycopeptides, with no resistance to vancomycin or teicoplanin detected. Resistance to ciprofloxacin was observed in one isolate (16.7%), while all isolates were susceptible to trimethoprim–sulfamethoxazole. No multidrug-resistant *S. aureus* isolates were identified.

Gram-negative bacteria were isolated only sporadically in infections associated with internal fixation devices and did not show clinically relevant resistance patterns. In particular, *Pseudomonas aeruginosa* isolates remained fully susceptible to carbapenems, and no extended-spectrum beta-lactamase–producing Enterobacterales or carbapenem-resistant organisms were detected.

Overall, antimicrobial resistance among bacterial isolates in infections associated with internal fixation devices was limited and characterized by a predominance of susceptible Gram-positive cocci.

## 4. Discussion

This monocentric retrospective study provides a focused description of the microbiological spectrum and antimicrobial resistance patterns of infections occurring in hand surgery, an area for which dedicated data remain limited. Most available evidence derives from broader orthopaedic or general surgical cohorts, often failing to capture the specific clinical and anatomical features of the hand. In addition to providing an updated snapshot (2024–2025), a key contribution of this study is the scenario-based stratification of microbiology and susceptibility patterns (trauma-related vs. internal fixation–associated infections) together with the distinction between monomicrobial and polymicrobial infections. This approach addresses a practical gap in the literature, where most data originate from broader orthopaedic or general surgical cohorts and do not fully reflect the specific anatomical, functional, and exposure-related features of the hand. As a result, empirical treatment in hand surgery is often extrapolated from non-hand settings, despite potentially relevant differences in microbial complexity and resistance epidemiology [21].

Trauma-related infections represented the most frequent clinical scenario and were commonly associated with polymicrobial involvement. This finding reflects the typical characteristics of traumatic hand injuries, including extensive tissue damage, environmental contamination, and delayed presentation, all of which increase the likelihood of polymicrobial colonization and infection [22,23]. Similar patterns have been reported in previous studies on open fractures and contaminated upper-limb injuries, where mixed aerobic and anaerobic flora are frequently identified [24].

In contrast, infections associated with internal fixation devices showed a more homogeneous microbiological profile, largely dominated by Gram-positive cocci, particularly *Staphylococcus aureus*. This finding is consistent with the well-established role of skin commensals and the ability of *S. aureus* to adhere to implanted materials and form biofilms, leading to persistent implant-associated infections [13,25,26,27].

Across all clinical scenarios, *Staphylococcus aureus* emerged as the leading pathogen in this cohort. This observation aligns with existing literature on hand and upper-extremity infections, in which *S. aureus* consistently represents the most frequently isolated microorganism, both in community-acquired and postoperative settings [28,29,30]. Monomicrobial infections were predominantly sustained by Gram-positive organisms, whereas polymicrobial infections exhibited greater microbiological diversity, including Gram-negative bacilli and anaerobic bacteria. Importantly, the present findings reinforce that microbiological complexity does not necessarily imply higher resistance pressure, supporting stewardship-oriented choices and limiting routine escalation to broad-spectrum regimens in the absence of specific risk factors [31].

The presence of Gram-negative bacteria, such as *Pseudomonas aeruginosa* and Enterobacterales, particularly in polymicrobial trauma-related infections, is consistent with contamination-related mechanisms and environmental exposure [32]. Anaerobic organisms, although less frequently isolated, have been described as relevant contributors in crush injuries and deep space infections of the hand, further supporting the need for adequate microbiological sampling in complex traumatic cases [33].

Overall, the burden of antimicrobial resistance observed in this study was limited. Methicillin resistance was mainly detected among *S. aureus* isolates from trauma-related infections, while infections associated with internal fixation devices showed low resistance rates. Notably, no ESBL-producing Enterobacterales or carbapenem-resistant organisms were identified. Although no high-level Gram-negative resistance phenotypes were detected, the total number of Gram-negative isolates in this cohort was limited. Therefore, these findings should be interpreted cautiously and cannot exclude the presence of such resistance phenotypes in larger populations. This absence of high-alert Gram-negative resistance provides a relevant local benchmark and contrasts with trends reported in other high-burden orthopaedic contexts, where MDR Gram-negative organisms are increasingly described in complex or revision implant-related infections [7,18,19].

From a clinical perspective, these results support a targeted empirical antimicrobial approach in hand surgery, primarily focused on Gram-positive coverage, with broader-spectrum regimens reserved for selected high-risk scenarios, such as severe trauma or polymicrobial infections. This approach is in line with current recommendations for antimicrobial stewardship and the management of musculoskeletal infections, emphasizing the importance of local epidemiology to guide empirical therapy [34]. In parallel, non-antibiotic adjunctive strategies are being explored to limit selective pressure, with in vitro studies reporting antimicrobial activity of silver-based compounds against multidrug-resistant skin pathogens [35].

From a broader clinical perspective, the impact of hand surgery infections extends beyond local surgical outcomes. Patients undergoing hand procedures frequently present with cardiovascular and metabolic comorbidities, particularly diabetes mellitus, conditions known to increase susceptibility to infection, impair wound healing, and prolong recovery. In this context, effective infection control and appropriate empirical antimicrobial strategies may indirectly influence cardiovascular stability, hospitalization duration, and the overall clinical burden in vulnerable populations. Consequently, local microbiological surveillance in specialized surgical units may contribute not only to optimized infection management but also to the broader care of patients with complex cardiometabolic profiles. The present study was not designed to assess infection incidence but rather to characterize local microbiological patterns and antimicrobial resistance profiles, generating data directly applicable to clinical decision-making in a specialized hand surgery setting. The main limitations include the retrospective design and the monocentric nature of the cohort, which may restrict generalizability. Nevertheless, given the limited availability of hand surgery–specific microbiological data in the literature, these findings provide a pragmatic unit-level reference for empirical therapy, particularly by quantifying the relative contribution of trauma-related versus fixation-associated infections and demonstrating that high-level resistance remains uncommon despite frequent polymicrobial involvement. Future multicenter investigations are warranted to confirm external validity and to monitor temporal trends in pathogen distribution and antimicrobial resistance in this specialized setting, in line with international recommendations emphasizing continuous local surveillance to guide empirical antimicrobial strategies [36]. The focus on principal isolates may underestimate resistance patterns among secondary co-isolated organisms, representing an inherent methodological limitation. In addition, data regarding prior antibiotic exposure were inconsistently documented in retrospective records and could not be reliably analyzed, potentially influencing culture yield and observed resistance profiles. Finally, clinical outcome variables such as reoperation rate, amputation, or length of hospitalization were not systematically captured in the microbiological database and were outside the predefined scope of the study, which was centered on pathogen distribution and resistance characterization. Because the total number of surgical procedures performed during the study period was not systematically captured, reliable SSI incidence estimates cannot be derived. The reported SSI proportion therefore reflects the distribution within the infected cohort rather than a population-level rate.

## 5. Conclusions

This study demonstrates that hand surgery infections are characterized by distinct microbiological patterns depending on the underlying clinical scenario. Trauma-related infections are frequently polymicrobial and microbiologically heterogeneous, whereas infections associated with internal fixation devices are predominantly monomicrobial and sustained by Gram-positive cocci, particularly *Staphylococcus aureus*.

Overall antimicrobial resistance levels were low, with limited methicillin resistance and no detection of ESBL-producing or carbapenem-resistant organisms. Despite the frequent occurrence of polymicrobial infections, high-level antimicrobial resistance remained uncommon across all subgroups.

These findings underline the importance of local microbiological surveillance in hand surgery and support a targeted, scenario-based approach to empirical antimicrobial therapy. By providing hand surgery–specific data in an area where the literature is limited, this study contributes valuable information to support clinical decision-making and antimicrobial stewardship in specialized upper limb units.

## Figures and Tables

**Table 1:** Demographic and clinical characteristics of study participants (*n* = 72).

Characteristics	Values
Age (years), mean ± standard deviation	56.8 ± 18.9
Gender	
Male	53 (73.6%)
Female	19 (26.4%)
Type of infection	
Trauma-related infections	56 (77.8%)
Infections associated with internal fixation	16 (22.2%)
Surgical site infections (SSI)	13 (18.1%)
Comorbidities	
Hypertension	3 (4.2%)
Diabetes mellitus	4 (5.6%)
Cardiovascular disease	4 (5.6%)
Smoking history	4 (5.6%)
Chronic obstructive pulmonary disease (COPD)	2 (2.8%)
Chronic kidney disease	2 (2.8%)
Systemic sclerosis/Sjögren syndrome	2 (2.8%)
Microbiological data	
Total bacterial isolates	105

**Table 2:** Distribution of microorganisms in monomicrobial and polymicrobial trauma-related infections (*n* = 56).

Trauma Infections (*n* = 56)	Monomicrobial	*n* = 36	Polymicrobial	*n* = 20
Microorganism	*Staphylococcus aureus* (MSSA)	16	*Staphylococcus aureus* (MSSA)	5
	*Staphylococcus aureus* (MRSA)	7	*Pseudomonas aeruginosa*	2
	*Staphylococcus epidermidis*	1	*Klebsiella oxytoca*	2
	*Streptococci* spp.	4	Enterobacterales	3
	*Pseudomonas aeruginosa*	2	Enterococci	2
	*Escherichia coli*	2	*Streptococci* spp.	3
	Others	4	Others	3

Abbreviations: MSSA, methicillin-susceptible *Staphylococcus aureus*; MRSA, methicillin-resistant *Staphylococcus aureus*.

**Table 3:** Distribution of microorganisms in monomicrobial and polymicrobial infections associated with internal fixation devices (*n* = 16).

Fixation-Related Infections (*n* = 16)	Monomicrobial	*n* = 9	Polymicrobial	*n* = 7
Microorganism	*Staphylococcus aureus* (MSSA)	5	*Staphylococcus aureus* (MSSA)	2
	*Staphylococcus aureus* (MRSA)	1	*Enterococcus faecalis*	1
	*Streptococci* spp.	2	Others	4
	*Pseudomonas aeruginosa*	1		

Abbreviations: MSSA, methicillin-susceptible *Staphylococcus aureus*; MRSA, methicillin-resistant *Staphylococcus aureus*.

**Table 4:** Antimicrobial resistance patterns of predominant bacterial isolates in trauma-related infections.

Antibiotic Agent	*Staphylococcus aureus* (*n* = 28) (%)	*Pseudomonas aeruginosa* (*n* = 4) (%)
Amoxicillin–clavulanate	–	–
Piperacillin–tazobactam	–	25.0
Ceftazidime	–	25.0
Cefepime	–	25.0
Imipenem	–	0
Meropenem	–	0
Ciprofloxacin	21.4	25.0
Trimethoprim–sulfamethoxazole	14.3	–
Oxacillin	25.0 (MRSA)	–
Vancomycin	0	–
Teicoplanin	0	–
MDR	3.6	25.0

Abbreviations: MRSA, methicillin-resistant *Staphylococcus aureus*; MDR, multidrug resistance.

## Data Availability

The data that support the findings of this study are not openly available due to reasons of sensitivity and are available from the corresponding author upon reasonable request. Data are located in controlled access data storage at Multimedica IRCCS.

## Supplementary Materials

The following supporting information can be downloaded at: https://www.mdpi.com/article/10.3390/pathogens15020183/s1, Table S1: Distribution and susceptibility summary of less frequent microorganisms isolated from hand surgery infections.
