# Supplementary material for: Microbiological Spectrum and Antimicrobial Resistance Patterns in Hand Surgery Infections: A Monocentric Retrospective Study
Source: Pathogens. 2026 Feb 6;15(2):183. doi: 10.3390/pathogens15020183 (PMC12942673; doi:10.3390/pathogens15020183)
Supplement: Supplementary file 1 [file pathogens-15-00183-s001.zip › pathogens-4140239-supplementary.pdf]

Microorganism	No. isolates	Susceptibility summary in this cohort
Leclercia adecarboxylata	1	Pan-susceptible isolate
Citrobacter freundii	2	No ESBL/carbapenem resistance detected
Citrobacter koseri	1	Pan-susceptible isolate
Enterobacter aerogenes	2	No ESBL/carbapenem resistance detected
Enterobacter cloacae complex	2	No ESBL/carbapenem resistance detected
Enterobacter asburiae	1	Pan-susceptible isolate
Klebsiella oxytoca	4	Pan-susceptible isolates; no ESBL detected
Klebsiella pneumoniae	1	Pan-susceptible isolate
Proteus mirabilis	2	Pan-susceptible isolates
Morganella morganii (ssp. *morganii/sibonii*)	2	No ESBL/carbapenem resistance detected
Serratia marcescens	3	No ESBL/carbapenem resistance detected
Pseudomonas putida	1	No carbapenem resistance detected
Pseudomonas aeruginosa	6	One MDR isolate; others susceptible or increased exposure per EUCAST
Stenotrophomonas maltophilia	1	Expected intrinsic resistance profile; managed per EUCAST
Anaerobes (*Prevotella spp.*, *Fusobacterium nucleatum*, *Bacteroides stercoris*)	10	No carbapenem resistance detected
Fungi (*Candida spp.*, *Aspergillus niger*)	3	Rare co-isolates; interpreted clinically

Table S1. Distribution and susceptibility summary of less frequent microorganisms isolated from hand surgery infections:

Supplementary Table S1 summarizes all microorganisms grouped under the "Others" category in the main tables, including uncommon aerobic bacteria, anaerobes, and fungi. For each organism, the number of isolates and a cohort-level susceptibility summary are reported according to routine EUCAST interpretation. No extended-spectrum β-lactamase (ESBL) production or carbapenem resistance was detected among Enterobacterales isolates. Anaerobic and fungal isolates were interpreted in the clinical context of polymicrobial infections. This table provides transparency regarding rare isolates while preserving readability of the main results tables.
